# Amyloid formation reduces protein kinase B phosphorylation in primary islet β-cells which is improved by blocking IL-1β signaling

**DOI:** 10.1371/journal.pone.0193184

**Published:** 2018-02-23

**Authors:** Yun Zhang, Garth L. Warnock, Ziliang Ao, Yoo Jin Park, Nooshin Safikhan, Aziz Ghahary, Lucy Marzban

**Affiliations:** Department of Surgery, Faculty of Medicine, University of British Columbia, Vancouver, BC, Canada; Baylor College of Medicine, UNITED STATES

## Abstract

Amyloid formation in the pancreatic islets due to aggregation of human islet amyloid polypeptide (hIAPP) contributes to reduced β-cell mass and function in type 2 diabetes (T2D) and islet transplantation. Protein kinase B (PKB) signaling plays a key role in the regulation of β-cell survival, function and proliferation. In this study, we used human and hIAPP-expressing transgenic mouse islets in culture as two *ex vivo* models of human islet amyloid formation to: 1. Investigate the effects of amyloid formation on PKB phosphorylation in primary islet β-cells; 2. Test if inhibition of amyloid formation and/or interleukin-1β (IL-1β) signaling in islets can restore the changes in β-cell phospho-PKB levels mediated by amyloid formation. Human and hIAPP-expressing mouse islets were cultured in elevated glucose with an amyloid inhibitor (Congo red) or embedded within collagen matrix to prevent amyloid formation. To block the IL-1β signaling, human islets were treated with an IL-1 receptor antagonist (anakinra) or a glucagon-like peptide-1 agonist (exenatide). β-cell phospho-PKB levels, proliferation, apoptosis, islet IL-1β levels and amyloid formation were assessed. Amyloid formation in both cultured human and hIAPP-expressing mouse islets reduced β-cell phospho-PKB levels and increased islet IL-1β levels, both of which were restored by prevention of amyloid formation either by the amyloid inhibitor or embedding islets in collagen matrix, resulting in improved β-cell survival. Furthermore, inhibition of IL-1β signaling by treatment with anakinra or exenatide increased β-cell phospho-PKB levels, enhanced proliferation and reduced apoptosis in amyloid forming human islets during 7-day culture. These data suggest that amyloid formation leads to reduced PKB phosphorylation in β-cells which is associated with elevated islet IL-1β levels. Inhibitors of amyloid or amyloid-induced IL-1β production may provide a new approach to restore phospho-PKB levels thereby enhance β-cell survival and proliferation in conditions associated with islet amyloid formation such as T2D and clinical islet transplantation.

## Introduction

Islet amyloid polypeptide (IAPP; amylin) [1, 2] is a 37-amino acid peptide hormone that is normally produced and secreted along with insulin from islet β-cells [3]. In soluble form, IAPP reduces food intake and plays a physiological role in the regulation of postprandial glycaemia by suppression of glucagon release and inhibition of gastric emptying [4]. However, human IAPP (hIAPP) aggregates are toxic to β-cells [5–8] and contribute to progressive β-cell dysfunction and death in type 2 diabetes (T2D) [4, 9–11] as well as in cultured [6–8] and transplanted islets [12–15]. It is not clear why soluble hIAPP molecules form non-soluble toxic aggregates in T2D but it appears that increased hIAPP production, presence of an amyloidogenic sequence, and impaired prohIAPP processing, all contribute to hIAPP aggregation [4, 16].

The mechanisms underlying amyloid-induced β-cell death *in vivo* are still not well understood, but *in vitro* studies suggest that multiple mechanisms contribute to amyloid-induced β-cell apoptosis [17–21]. Moreover, a previous study has shown that replicating β-cells are more susceptible to amyloid-induced cytotoxicity [22], raising the idea that amyloid formation may result in failure to adaptive increase in β-cell mass in patients with T2D. While mechanisms that mediate β-cell toxic effects of hIAPP aggregates have been extensively studied, our current knowledge about the effects of hIAPP aggregates on β-cell proliferation is very limited.

Growing evidence from our studies and those of others suggest that interleukin 1β (IL-1β) signaling is an important mediator of amyloid-induced β-cell death in cultured and transplanted islets [7, 8, 23–26]. Amyloid-induced IL-1β production may also contribute to islet inflammation and β-cell death in T2D [27, 28]. Protein kinase B (PKB or Akt) signaling pathway plays a key role in the regulation of β-cell survival, function and proliferation, both *in vitro* and *in vivo* [29–32]. In the present study, we used islets from cadaveric pancreatic donors and transgenic mice with β-cell-specific hIAPP expression, to examine the effects of endogenously formed hIAPP aggregates on PKB phosphorylation in primary islet β-cells. We further tested if inhibition of amyloid formation (by an amyloid inhibitor or embedding islets in collagen matrix) and/or pharmacological inhibition of amyloid-induced IL-1β signaling can restore alterations in β-cell phospho-PKB levels mediated by amyloid formation in islets.

## Materials and methods

### Culture of human islets

Human islets for this study were isolated from cadaveric pancreatic donors by Ike Barber Human Islet Transplant Laboratory (Vancouver, BC, Canada) in accordance with the approved procedures and guidelines by Health Canada and the Clinical Research Ethics Board of the University of British Columbia. None of the cadaveric pancreatic donors were from a vulnerable population and all donors or next of kin provided written informed consent for use in research and education. Also, all research protocols for human islets used in this study were approved by the Clinical Ethics Board of the University of British Columbia. Isolated human islets (purity ~90% as assessed by dithizone staining) were cultured at different conditions: free-floating in non-adherent culture plates in Ham’s/F10 (Invitrogen, Burlington, ON, CA) or CMRL (Mediatech, Herndon, VA, USA), with the amyloid-binding dye Congo red (25 μmol/L; Sigma-Aldrich, Oakville, ON, CA), exenatide (Byetta; 10nmol/L; Amylin Pharmaceuticals, San Diego, CA, USA) or anakinra (Kineret; 10μg/mL; Sobia Pharmaceutics, Denton, MD, USA). For three-dimensional scaffold studies, human islets were embedded within collagen matrix / Ham’s-F10 as described before [33]. Culture medium was supplemented with 11.1 mmol/L glucose, 50 U/mL penicillin, 50 μg/mL streptomycin and 50 μg/mL gentamicin, 0.5% (w/v) BSA (Ham’s/F10) or 10% (vol./vol.) FBS (CMRL). Islets were cultured for 7 days in a humid atmosphere (95% air, 5% CO_2_) at 37°C and medium was replaced every 48 hours.

### Animal model

Hemizygous C57BL/6 hIAPP transgenic mice (hIAPP^+/-^) with β-cell specific hIAPP expression were kindly provided by Dr. S. Kahn (University of Washington, Seattle, WA, USA) and maintained by breeding with DBA/2J mice (Jackson Laboratory, Bar Harbor, ME, USA). Male hIAPP^+/-^ mice on high fat diet form islet amyloid *in vivo* and develop amyloid-associated diabetes in about one year [34], whereas isolated hIAPP^+/-^ mouse islets form islet amyloid within days during culture in high glucose *in vitro* [7, 35]. Mice were fed a chow containing 9% (w/w) fat (Purina 5021; LabDiet, Richmond, IN, USA). Animals were cared for in accordance with the Guidelines and Principles of Laboratory Animal Care, and the standard procedures established by the Canadian Council on Animal Care and the University on British Columbia’s Animal Policy and Welfare Committee. All research protocols used in these animal studies were approved by the University of British Columbia Animal Care and User Committee.

### Mouse islet isolation and culture

Wild-type (hIAPP^-/-^) and transgenic (hIAPP^+/-^) mice (8–12 weeks) were anaesthetized with tribromoethanol (0.25 mg/g body weight, i.p.), terminated by cervical dislocation, and then pancreatic islets were isolated as described before [8]. Briefly, pancreases were distended through the pancreatic duct with 2 mL calcium-free Hanks’ buffer containing 1000 U/mL of ice-cold collagenase (Type XI; Sigma-Aldrich). The distended pancreases were then removed and incubated at 37°C for 14 min with addition of 2 mL collagenase (1,000 U/mL in Hanks’ buffer), followed by gentle shaking for 2 min. Digestion was stopped by adding ice-cold Hanks’ buffer containing 1 mmol/L CaCl_2_. Islets were purified by passing the digested pancreatic tissue through 70 μm mesh cell strainers (BD Biosciences, Oakville, ON, CA). Hand-picked islets (purity >95%) were cultured overnight to allow recovery and then cultured in non-coated 48-well plates (50 islets/well) under three different conditions: free-floating in Ham’s-F10 medium, free-floating in Ham’s-F10 medium with Congo red (CR; 25 μmol/l) or embedded within collagen matrix/Ham’s-F10. Islets were cultured for 7 days in a humid atmosphere (95% air, 5% CO_2_) at 37°C. Ham’s-F10 medium was supplemented with 16.7 mmol/L glucose, 0.5% (w/v) BSA and antibiotics as described for human islets.

### Immunohistochemistry, TUNEL assay and thioflavin S staining

Paraffin-embedded sections (5 μm) of islets were dewaxed, rehydrated and blocked in 2% normal goat and/or donkey serum (Vector Laboratories, Burlingame, CA, USA). Fixed islet sections (following antigen retrieval with citrate buffer) were incubated at 4°C overnight with guinea pig anti-insulin alone (1:750; Dako, Carpinteria, CA, USA) or with one of the following: rabbit anti-phospho-PKB (1:100; Cell Signaling, Pickering, ON, Canada), anti-oligomer (A11; 1:400; Invitrogen), anti-IL-1β (1:100; Santa Cruz, Santa Cruz, CA, USA), anti-glucagon (1:1000; Sigma-Aldrich) or anti-PCNA (1:250; Cell Signaling). Islet sections were then incubated with Texas red-conjugated (or AMCA-conjugated) anti-guinea pig (Jackson Laboratories, West Grove, PA, USA) alone or with Alexa 488-conjugated anti-rabbit (Molecular Probes, Eugene, OR, USA) for 1 h at room temperature. Islet sections immunolabelled for insulin and A11 oligomers were incubated with Alexa 488-conjugated anti-guinea pig (Molecular Probes) and Texas red-conjugated anti-rabbit (Jackson). For double insulin and TUNEL or thioflavin S staining, after immunolabelling for insulin, islet sections were incubated with TUNEL reaction mixture (Roche Diagnostics, Laval, QC, Canada) for 30 min at 37°C or incubated with 0.5% (w/v) thioflavin S solution (Sigma-Aldrich) for 5 min at room temperature. Thioflavin S staining and A11 immunolabeling were used to detect large and small (oligomer) hIAPP aggregates in islets, respectively. The details of antibodies are summarized in S1 Table.

### Quantitative analysis of micrographs

Islet β-cell apoptosis and proliferation were reported as the percentage of double insulin and TUNEL-positive or PCNA-positive islet cells to total number of islet cells, respectively. The proportions of amyloid (thioflavin S)-positive and A11 (oligomer)-positive islets were calculated as the percentage of thioflavin S or oligomer-positive islets to total number of islets, respectively. Islet amyloid area was reported as the percentage of thioflavin S positive islet area to total islet area. Intensity of phospho-PKB immunofluorescence in insulin-positive islet areas was quantified by Image-Pro analyzer software (version 6.3; Media Cybernetics, MD, USA) and reported as fold over wild-type (for hIAPP^+/-^ mice) or fold over day 0 (for human islets). Quantifications were performed on 25–30 islets per condition from 5 human islet donors (n = 3 for collagen matrix studies) or wild-type and hIAPP^+/-^ mice (3 mice/group in each study; 3 independent studies). Phospho-PKB quantifications were performed on amyloid-positive islets in each condition (to assess the effects of amyloid formation on PKB phosphorylation) and an equal number of islets from amyloid-negative conditions (as control) in a total of 15–20 human or hIAPP^+/-^ mouse islets per condition as detailed in each figure legend. To assess the impact of phospho-PKB changes in amyloid-positive islets on the overall islet β-cell survival rate in each condition, β-cell apoptosis and proliferation were quantified in all islets.

### Islet IL-1β release

The culture medium was collected on day 4 and centrifuged (12,000 g, 10 min, 4°C) to remove cell debris. The supernatants were frozen at -20°C until assayed. IL-1β in the culture medium was measured using a human specific IL-1β ELISA kit (R & D Systems, Minneapolis, MN, USA). The culture medium alone (without islets) was used as control for background reading.

### Electrophoresis and immunoblotting

About 100 human islets were lysed in 30 μL lysis buffer containing 50 mmol/L Tris-HCl (pH 8.0), 150 mmol/L NaCl, 0.02% sodium azide, 0.1% sodium dodecyl sulphate, 1% Nonidet P-40, 0.5% sodium deoxycholate, 1 mmol/L PMSF and 10 μg/ml aprotinin for 25 min on ice and vortexed every 5 min. Samples were centrifuged (15,000 g, 10 min, 4°C) and the supernatant fractions were frozen at **−**70°C until analyzed. Aliquots of protein (15μg) from islet lysates were electrophoresed on polyacrylamide gel, then incubated for 1 h at room temperature with rabbit anti-phospho-PKB (1:750; Cell Signaling) which detects Ser473 phosphorylated PKB (or its equivalent sites on PKBβ-Ser474 and PKBγ-Ser472) or rabbit anti-total PKB (1:750; Cell Signaling) which detects all three forms of PKB. Membranes were then washed and incubated with horseradish peroxidase-conjugated anti-mouse IgG (1:5000; Amersham, Baie D’Urfe, QC, CA) for 1 h. Immunodetection was performed using an enhanced chemiluminescence detection kit (Amersham). Protein bands on the films were analyzed by densitometry using Image Lab software (Bio-Rad, Mississauga, ON, CA).

### Statistical analysis

Data are expressed as means ± SEM. Statistical analyses were performed using Student’s *t*-test or one-way analysis of variance (one-way ANOVA), followed by post hoc multiple comparison test. *P < 0*.*05* was considered statistically significant.

## Results

### Biosynthetic hIAPP aggregates reduce β-cell phospho-PKB levels and proliferation rate in human and hIAPP-expressing transgenic mouse islets, both of which are prevented by the amyloid inhibitor Congo red

**Fig 1:**
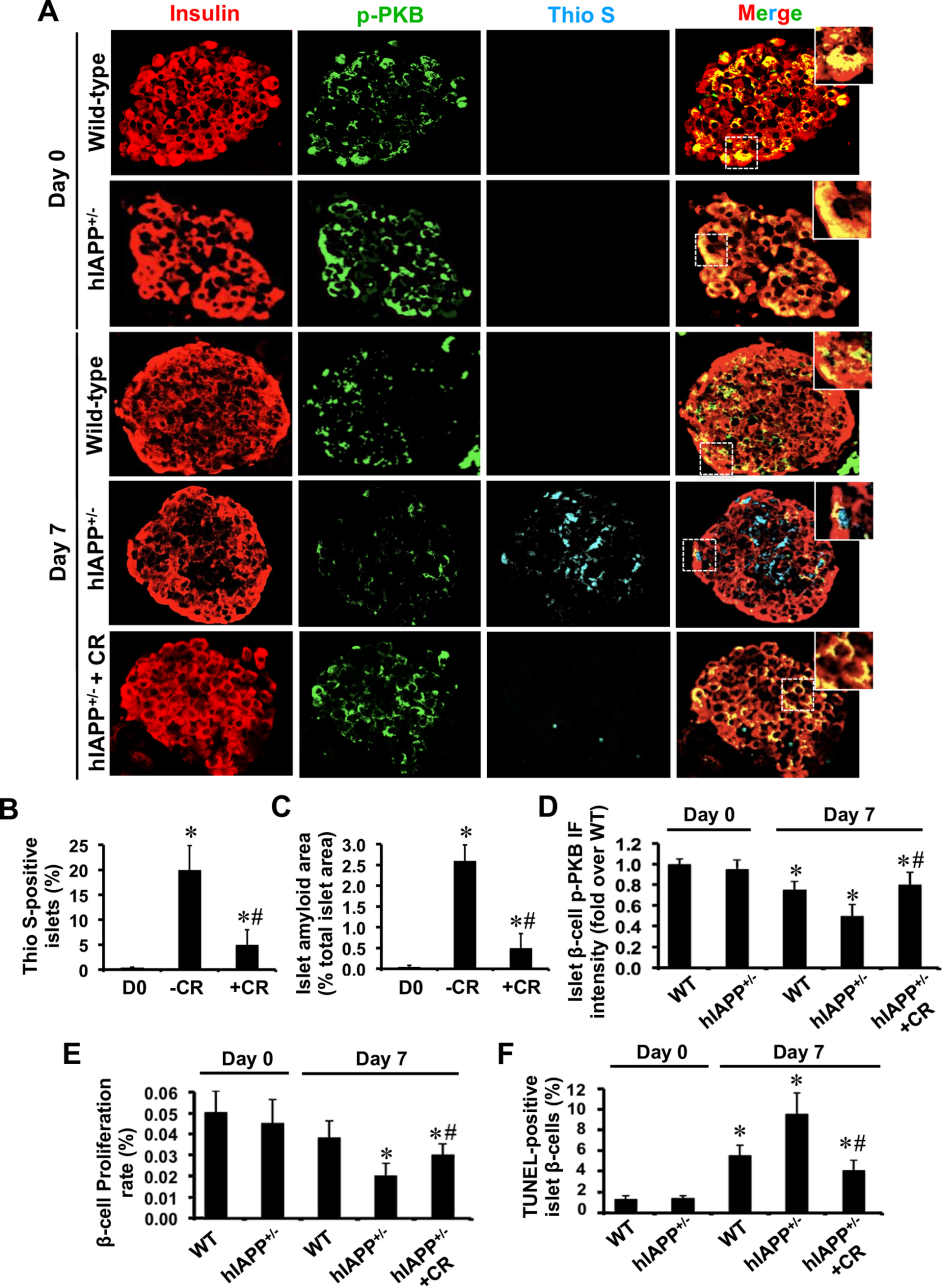
Formation of hIAPP aggregates is associated with reduced β-cell phospho-PKB levels and proliferation rate in hIAPP^+/-^ transgenic mouse islets during culture. **(A)** Paraffin-embedded islet sections from freshly isolated and 7-day cultured hIAPP^-/-^ (wild-type) and hIAPP^+/-^ mice with or without the amyloid binding dye Congo red (CR; 25 μmol/L), were immunolabelled for insulin, phospho-PKB (p-PKB), and thioflavin S (Thio S). The squares (dashed white lines) correspond to enlarged areas in each image (original magnification: X400; insert: X1000). The percentage of **(B)** thioflavin S (amyloid)-positive islets and **(C)** islet amyloid area. **(D)** β-cell phospho-PKB immunofluorescence (IF) intensity. The percentage of **(E)** PCNA-positive (proliferative) β-cells and **(F)** TUNEL-positive (apoptotic) β-cells in each condition. Results are expressed as mean +/- SEM of three independent studies (25–30 islets per condition from n = 3 mice per group in each group). For β-cell phospho-PKB IF intensity, quantifications were performed on a total of 20 amyloid (thio S)-positive 7-day cultured hIAPP^+/-^ islets and equal number of WT islets (amyloid-negative) or CR-treated hIAPP^+/-^ islets (no or very little amyloid formation). *vs Day 0; **#**vs corresponding untreated group (*P<0*.*05*; one-way ANOVA).

**Fig 2:**
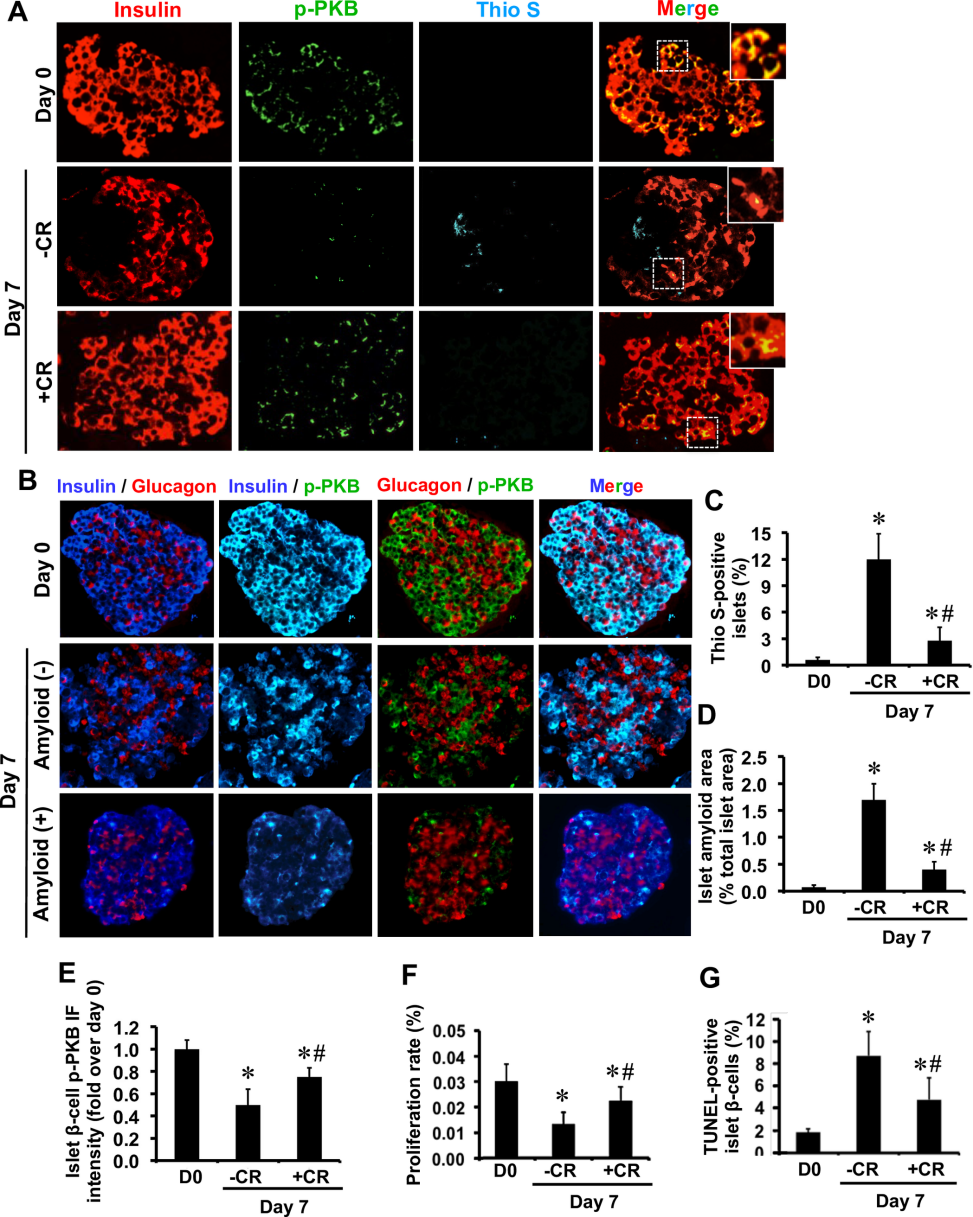
Progressive amyloid formation in human islets during culture is associated with reduced β-cell phospho-PKB levels and proliferation rate. **(A)** Paraffin-embedded sections from pre-culture and 7-day cultured human islets with or without amyloid binding dye Congo red (CR; 25 μmol/L) were immunolabelled for insulin, phospho-PKB (p-PKB), and thioflavin S (Thio S). The squares (dashed white lines) correspond to enlarged areas in each image (original magnification: X400; insert: X1000). **(B)** Immunolabelling for insulin or glucagon and phospho-PKB in amyloid-positive and negative human islets. The percentage of **(C)** thioflavin S (amyloid)-positive islets and **(D)** islet amyloid area. **(E)** Islet phospho-PKB immunofluorescence intensity (IF). The proportion of **(F)** PCNA-positive (proliferative) β-cells and **(G)** TUNEL-positive (apoptotic) β-cells in each condition. Results are expressed as mean +/- SEM of five independent studies (25–30 islets per condition in each study). For β-cell phospho-PKB IF intensity, quantifications were performed on a total of 18 amyloid (thio S)-positive 7-day cultured human islets or equal number of CR-treated islets (no or very little amyloid formation). *vs Day 0; **#**vs corresponding untreated group *(P<0*.*05*; one-way ANOVA).

We examined the effects of biosynthetic hIAPP aggregates on PKB phosphorylation in primary islet β-cells. Isolated human or wild-type and hIAPP^+/-^ transgenic mouse islets were cultured for 7 days in elevated glucose (to potentiate amyloid formation) in the presence or absence of the amyloid inhibitor Congo red. Freshly isolated hIAPP^+/-^ transgenic mouse islets did not contain amyloid but they formed amyloid during 7-day culture (Fig 1A–1C) which correlated with their lower β-cell phospho-PKB levels as compared to wild-type mouse islets (Fig 1A and 1D). Similarly, amyloid formation in human islets during 7-day culture (Fig 2A, 2C and 2D) was associated with reduced phospho-PKB levels in β-cells (Fig 2A and 2E), resulting in lower β-cell phospho-PKB levels in 7-day cultured thioflavin S (amyloid)-positive human islets than amyloid-negative islets (Fig 2B). The reduction in β-cell phospho-PKB levels in hIAPP^+/-^ transgenic mouse islets and human islets during amyloid formation correlated with the reduced rate of proliferation (Figs 1E and 2F) and increased rate of apoptosis (Figs 1F and 2G). Moreover, treatment with the amyloid inhibitor Congo red markedly decreased amyloid formation, restored β-cell phospho-PKB levels and enhanced β-cell survival in both human and hIAPP^+/-^ transgenic mouse islets (Figs 1 and 2).

### Reducing amyloid formation by embedding human or hIAPP^+/-^ transgenic mouse islets in collagen matrix restores phospho-PKB levels and decreases β-cell apoptosis

**Fig 3:**
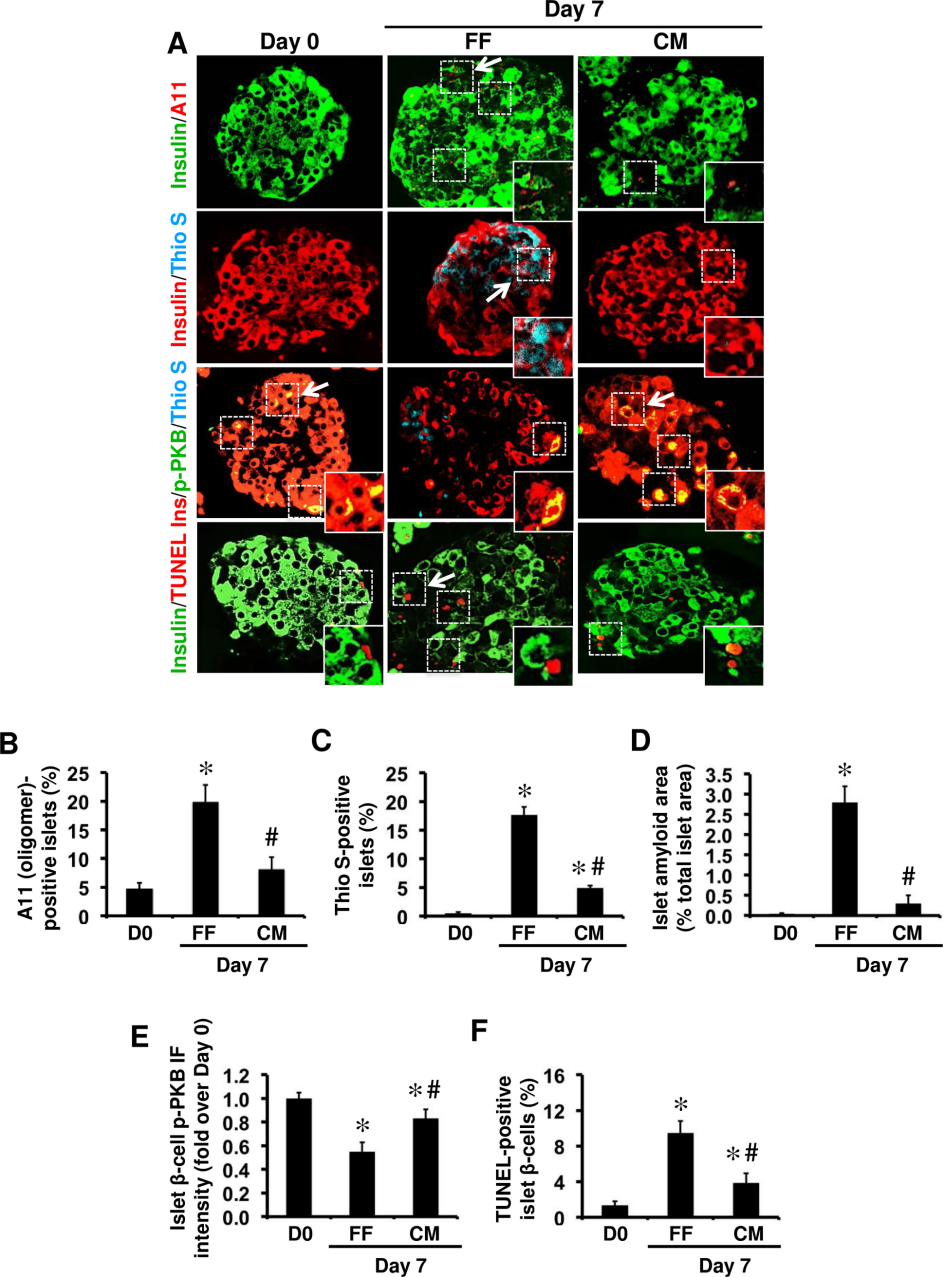
Collagen matrix-embedded hIAPP^+/-^ transgenic mouse islets have lower amyloid formation, β-cell apoptosis, and higher β-cell phospho-PKB levels than free-floating cultured islets. **(A)** Islet sections from hIAPP^+/-^ mice were immunolabelled for insulin/A11 (oligomer), insulin/thioflavin S (Thio S), insulin/p-PKB/Thio S or insulin/TUNEL before and after 7-day culture free-floating (FF) or in collagen matrix (CM). The arrows point to regions corresponding to enlarged areas in each image (original magnification: X400; insert: X1000). The percentage of **(B)** A11 (oligomer)-positive islets, **(C)** Thioflavin S (amyloid)-positive islets, and **(D)** islet amyloid area. **(E)** β-cell phospho-PKB immunofluorescence (IF) intensity. **(F)** The proportion of apoptotic β-cells. Results are expressed as mean +/- SEM of three independent studies (25–30 islets per condition from n = 3 mice per group in each study). For β-cell phospho-PKB IF intensity, quantifications were performed on a total of 15 amyloid (thio S)-positive 7-day cultured hIAPP^+/-^ islets and equal number of CM-embedded islets (no or very little amyloid formation). *vs Day 0; **#**vs FF, one-way ANOVA (*P<0*.*05*; one-way ANOVA).

We next cultured hIAPP^+/-^ transgenic mouse islets in elevated glucose either free-floating (to form amyloid) or embedded within three-dimensional collagen matrix (to reduce amyloid formation) [33]. Small hIAPP aggregates (oligomers) were detectable only in a low number (~5%) of pre-culture hIAPP^+/-^ mouse islets but following 7 days culture this ratio was increased to ~20% in free-floating islets which was much lower (~8%) in collagen matrix-embedded islets (Fig 3A and 3B). The number of amyloid-positive islets (Fig 3C) and islet amyloid area (Fig 3D) were also lower in 7-day cultured collagen matrix-embedded islets than free-floating islets. Interestingly, hIAPP^+/-^ transgenic mouse islets, in which amyloid formation was markedly reduced by embedding in collagen matrix had higher phospho-PKB levels (Fig 3E) and lower number of apoptotic β-cells (Fig 3F) than free-floating cultured islets with amyloid formation.

**Fig 4:**
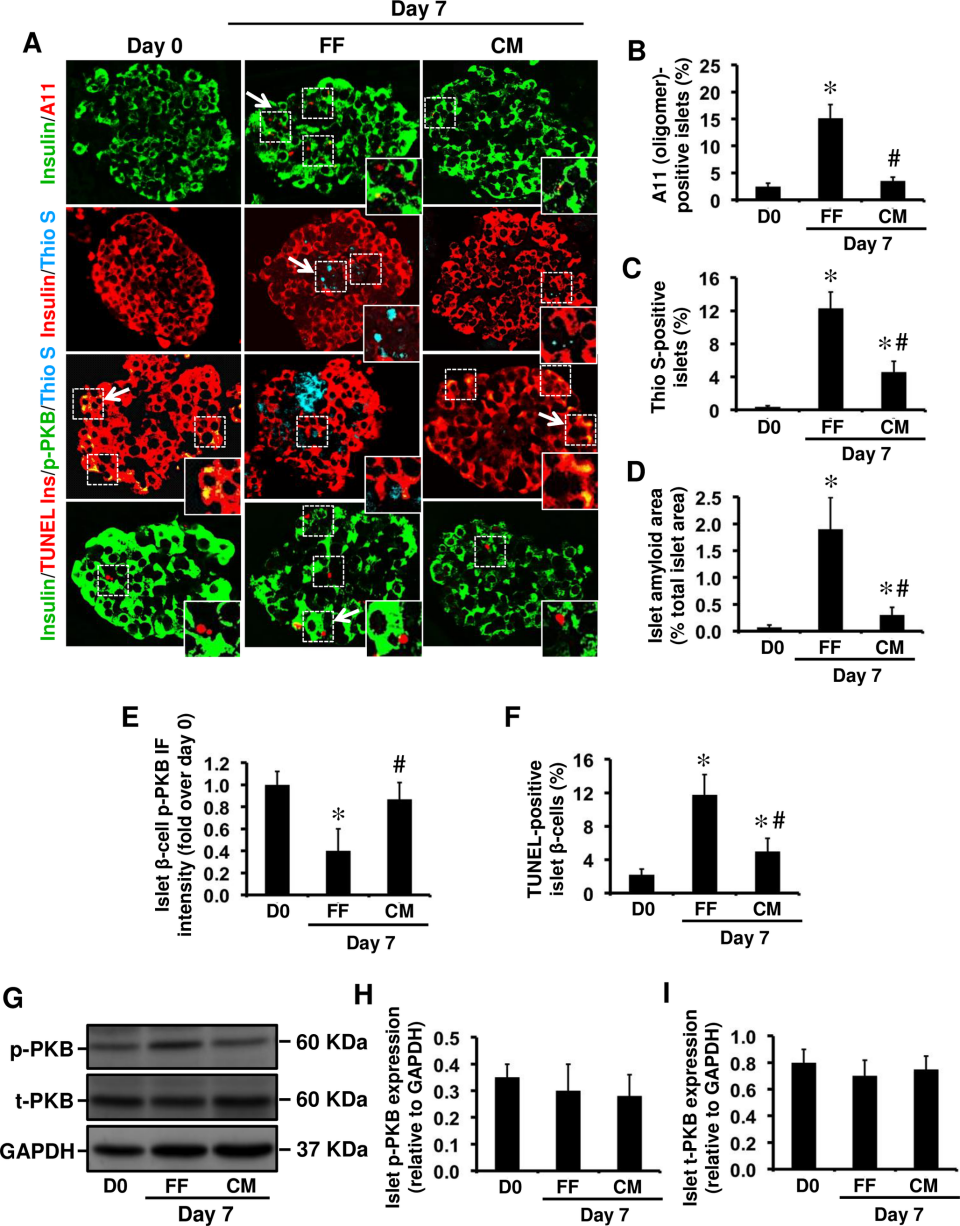
The lower amyloid formation in collagen matrix-embedded human islets than free-floating cultured islets correlates with higher phospho-PKB levels and enhanced β-cell survival. **(A)** Islet sections from pre-culture and 7-day collagen-matrix embedded (CM) and free-floating (FF) cultured human islets were immunolabelled for insulin/A11, insulin/thioflavin S (Thio S), insulin/p-PKB/Thio S or insulin/TUNEL. The arrows point to regions corresponding to enlarged areas in each image (original magnification: X400; insert: X1000). The percentage of **(B)** A11 (oligomer)-positive islets, **(C)** thioflavin S (amyloid)-positive islets, and **(D)** Islet amyloid area. **(E)** β-cell phospho-PKB immunofluorescence intensity. **(F)** The proportion of apoptotic β-cells. **(G-I)** Phospho-PKB (p-PKB) and total PKB (t-PKB) protein levels in whole islet lysates assessed by Western blot. Data are expressed as means ± SEM of three independent studies (30 islets per condition in each study). For β-cell phospho-PKB IF intensity, quantifications were performed on a total of 15 amyloid (thio S)-positive 7-day cultured human islets and equal number of CM-embedded islets (no or very little amyloid formation). *vs Day 0; **#**vs FF (*P<0*.*05*; one-way ANOVA).

Consistent with our findings in hIAPP^+/-^ transgenic mouse islets, reduced amyloid formation in 7-day cultured human islets embedded in collagen matrix (Fig 4A–4D) was associated with higher β-cell phospho-PKB levels (Fig 4E) and lower number of apoptotic β-cells (Fig 4F). There was no detectable difference between phospho-PKB levels in whole islet lysates from free-floating and collagen-matrix embedded human islets by Western blot (Fig 4G–4I). It is worth noting that human islet lysates contained both amyloid-positive (~10–15%) and amyloid-negative (~85–90%) islets, so the changes in β-cell phospho-PKB levels in amyloid-positive islets detected by immunolabelling are likely masked by the presence of amyloid-negative islets in whole islet lysates.

### Reduced phospho-PKB levels in amyloid forming human islets correlates with increased IL-1β levels

**Fig 5:**
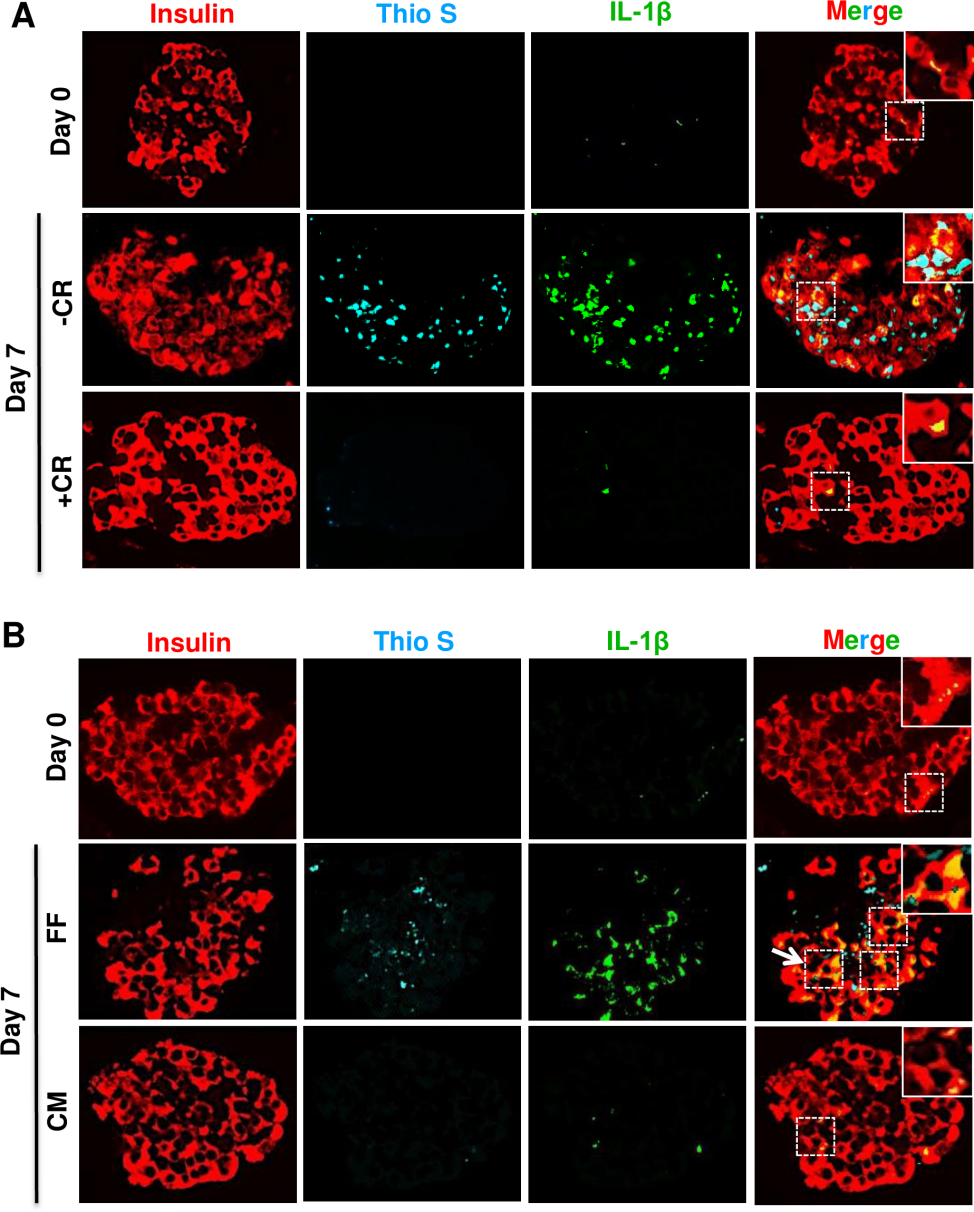
Restoring phospho-PKB levels in cultured human islets by prevention of amyloid formation is associated with reduced islet IL-1β levels. Paraffin-embedded sections from pre-culture and 7-day cultured human islets were **(A)** incubated with or without the amyloid-binding dye Congo red (CR; 25μmol/L) or **(B)** embedded in collagen matrix and immunolabelled for insulin, IL-1β, and Thio S. The squares (dashed white lines) correspond to enlarged areas in each image (original magnification: X400; insert: X1000). The micrographs are representative of three independent studies.

Interestingly, islet amyloid mediated decrease in phospho-PKB levels in β-cells (Figs 2 and 4) correlated with elevated islet IL-1β levels in cultured human islets (Fig 5). Furthermore, restoring β-cell phospho-PKB levels in human islets by prevention of amyloid formation either with Congo red (Fig 5A) or collagen matrix (Fig 5B) was associated with decreased islet IL-1β levels, suggesting a potential role for IL-1β in mediating amyloid-induced reduction of PKB phosphorylation.

### Inhibition of IL-1β signaling by anakinra or exenatide restores β-cell phospho-PKB levels and enhances islet survival in human islets during culture

**Fig 6:**
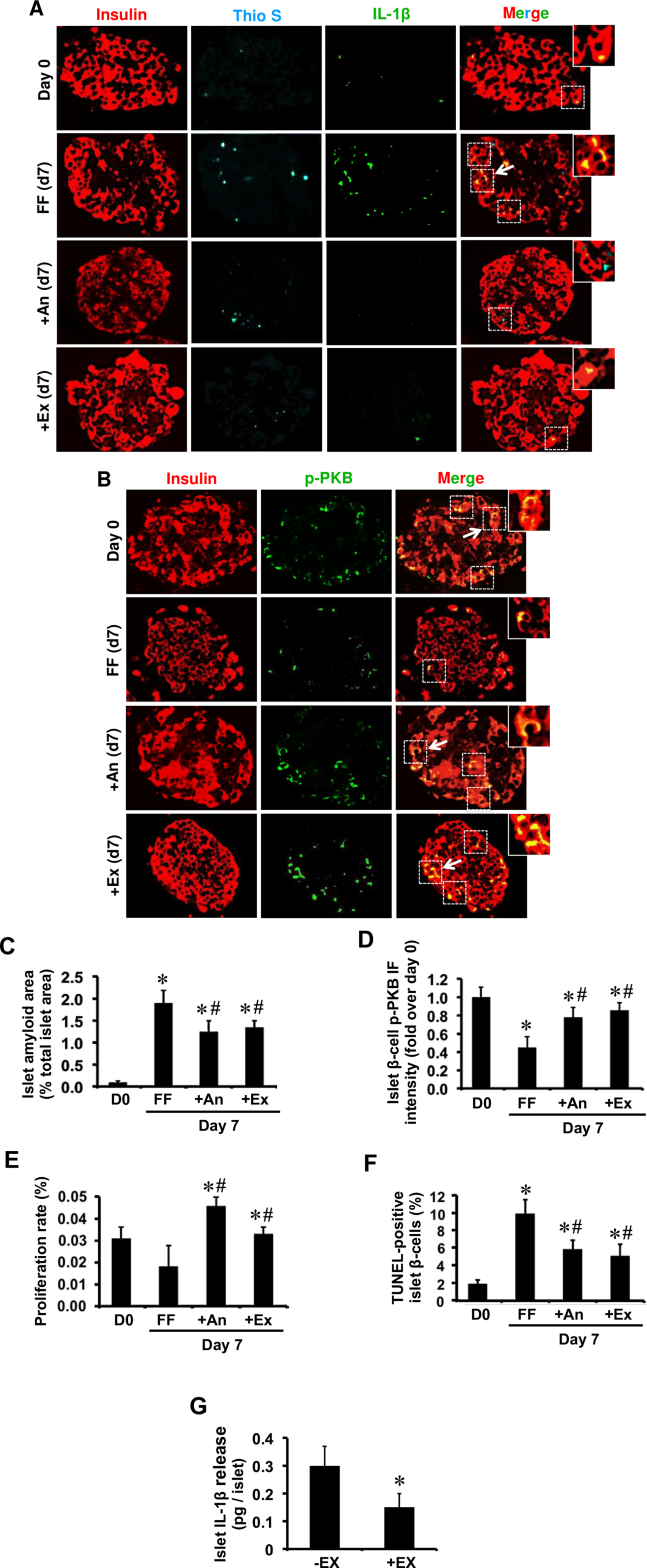
Treatment with anakinra or exenatide reduces IL-1β levels in human islets during culture which is associated with increased β-cell phospho-PKB levels, enhanced proliferation and reduced apoptosis. Islet sections from pre-culture and 7-day cultured human islets with anakinra (An; 10 μg/mL) or exenatide (Ex; 10 nmol/L) were immunolabelled for **(A)** insulin/IL-1β/Thio S or **(B)** insulin/p-PKB. **(C)** The percentage of islet amyloid area to total islet area in each condition. **(D)** β-cell phospho-PKB immunofluorescence (IF) intensity. The percentage of **(E)** PCNA-positive (proliferative) β-cells and **(F)** TUNEL-positive (apoptotic) β-cells. **(G)** Islet IL-1β release from 4-day cultured exenatide-treated and non-treated human islets. The arrows point to regions corresponding to enlarged areas in each image (original magnification: X400; insert: X1000). Results are expressed as mean +/- SEM of five independent studies (5 donors; 25–30 islets per condition in each study). For β-cell phospho-PKB IF intensity, quantifications were performed on a total of 20 amyloid-positive 7-day cultured human islets and equal number of anakinra- or exenatide-treated human islets (lower amyloid formation).*vs Day 0; **#**vs corresponding untreated group (*P<0*.*05*; one-way ANOVA or Student’s *t*-test).

We further tested whether pharmacological inhibition of IL-1β signaling can prevent hIAPP-induced reduction of phospho-PKB in islet β-cells. Freshly isolated human islets were cultured as detailed in the presence or absence of anakinra, a clinically approved IL-1 receptor antagonist. Pre-culture human islets with little or no detectable amyloid formation had low IL-1β levels (Fig 6A and 6C) and high phospho-PKB levels (Fig 6B and 6D). After 7-day culture, anakinra-treated human islets had markedly lower IL-1β immunoreactivity (Fig 6A), which was associated with higher phospho-PKB levels in β-cells as compared with non-treated human islets (Fig 6B and 6D). Similarly, human islets treated with exenatide, a clinically approved glucagon-like peptide-1 receptor agonist, had lower islet IL-1β immunoreactivity (Fig 6A), islet IL-1β release (Fig 6G) and higher β-cell phospho-PKB levels (Fig 6D) than non-treated cultured human islets. Finally, increased levels of phospho-PKB in exenatide and anakinra treated amyloid forming human islets was associated with enhanced β-cell proliferation (Fig 6E) and reduced β-cell apoptosis (Fig 6F).

## Discussion

While mechanisms of islet amyloid-induced β-cell apoptosis have intensively been investigated in the past decade, our current knowledge on the effects of amyloid formation on β-cell proliferation and the underlying signaling pathways are very limited. Less focus on the effects of hIAPP aggregates on β-cell proliferation is likely related to the notion that replication is a rare event in primary islet β-cells. Therefore, contribution of the changes in β-cell proliferation to reduced β-cell mass has been underestimated. However, growing evidence from recent studies have changed this notion by demonstrating proliferation of β-cells in human islets both *in vitro* and *in vivo* [36, 37]. The balance between β-cell proliferation and apoptosis is a key factor in the regulation of islet β-cell mass. Thus, chronic changes in β-cell proliferation in pathological conditions that are associated with increased β-cell apoptosis such as diabetes may play a significant role in the regulation of β-cell mass.

In the present study, using two *ex vivo* models of human islet amyloid formation, human and hIAPP^+/-^ transgenic mouse islets, we demonstrate that formation of biosynthetic hIAPP aggregates results in reduced PKB phosphorylation in β-cells possibly by promoting islet IL-1β production. We further show that inhibition of islet amyloid formation or pharmacological inhibition of IL-1β signaling improves decreased phospho-PKB levels mediated by amyloid formation, enhance β-cell proliferation and reduce β-cell apoptosis during *ex vivo* islet culture.

Human and hIAPP-expressing transgenic mouse islets were cultured in elevated glucose to form amyloid similar to that observed in patients with T2D [10] and human islet grafts in patients with T1D [12]. Formation of endogenously produced hIAPP aggregates in human islets during culture closely correlated with a marked decrease in β-cell phospho-PKB levels. Interestingly, amyloid-induced reduction in β-cell phospho-PKB levels was associated with elevated IL-1β levels in human islets. Similarly, islets from hIAPP^+/-^ transgenic mice that formed amyloid during culture had lower phospho-PKB levels than those from wild-type littermates which did not form amyloid. Taken together, these findings suggest that formation of biosynthetic hIAPP aggregates results in decreased phospho-PKB levels in primary islet β-cells.

We next examined if prevention of amyloid formation by embedding islets in collagen matrix or by treatment with an amyloid inhibitor can restore β-cell phospho-PKB levels. We previously showed that re-establishment of islet extracellular matrix by embedding islets in three-dimensional type 1 collagen matrix improves β-cell survival and function, resulting in a markedly lower amyloid formation in islets [33]. Interestingly, inhibition of amyloid formation in human islets either by amyloid inhibitor or by embedding in collagen matrix reduced islet IL-1β levels and restored β-cell phospho-PKB levels. Moreover, amyloid-induced reduction in phospho-PKB level was associated with decreased β-cell proliferation and increased β-cell apoptosis in human and hIAPP^+/-^ transgenic mouse islets, both of which were restored by prevention of amyloid formation.

**Fig 7:**
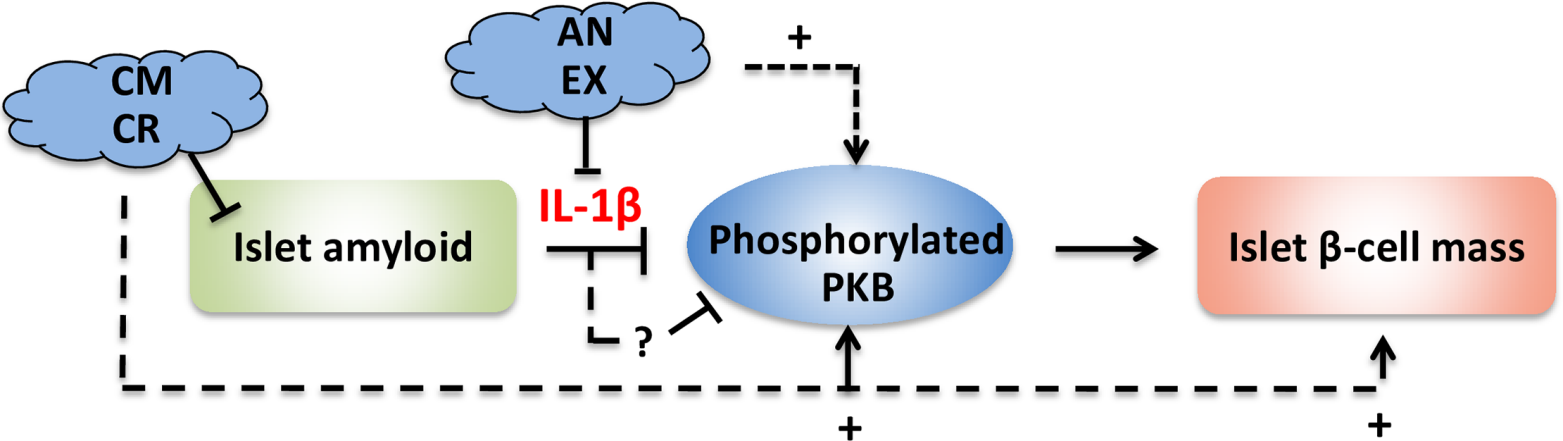
Proposed mechanism for amyloid-induced reduction in β-cell phospho-PKB levels and potential strategies to prevent this process. Amyloid formation leads to reduced β-cell phospho-PKB levels possibly by promoting IL-1β production in islets. Prevention of amyloid formation or blocking IL-1β signaling may provide two strategies to restore amyloid-induced decrease in phospho-PKB levels thereby improve islet β-cell mass. (CM: collagen matrix; CR: Congo red; Ex: Exenatide; An: Anakinra).

To further examine the role of IL-1β in mediating the effects of islet amyloid on phospho-PKB levels in primary islet β-cells, human islets were treated with anakinra, a clinically approved IL-1 receptor antagonist that competes with IL-1β for binding to IL-1 receptor 1 (IL-1R1), thereby blocking IL-1β signaling pathway. We found that anakinra-treated human islets had lower islet IL-1β levels, higher β-cell phospho-PKB levels, enhanced proliferation, and lower rate of apoptosis than non-treated cultured islets. Similarly, human islets treated with exenatide, a GLP-1 receptor agonist, had lower islet IL-1β immunoreactivity and IL-1β release which correlated with their higher β-cell phospho-PKB levels, proliferation and survival rates as compared to non-treated cultured human islets. Taken together, these findings suggest that formation of biosynthetic hIAPP aggregates leads to reduced PKB phosphorylation probably by promoting islet IL-1β production and that prevention of amyloid formation or blocking IL-1β signaling can improve amyloid-induced reduction in phospho-PKB levels, thereby enhancing β-cell proliferation and survival (Fig 7).

IL-1β has been shown to impair insulin signaling by targeting insulin receptor substrate-1 (IRS-1) and phosphoinositide 3-kinase (PI3K) in adipocytes [38, 39]. So one potential mechanism would be that IL-1β mediates amyloid-induced reduction in PKB phosphorylation in β-cells by impairing insulin signaling. Further mechanistic studies are required to identify the molecular mechanisms by which amyloid formation leads to reduced PKB phosphorylation in β-cells and the role of IL-1β in this process.

We recently showed that amyloid formation induces β-cell Fas upregulation and activation of the Fas-mediated apoptotic pathway via IL-1β signaling [7, 8, 26]. Thus, it appears that IL-1β plays a role in mediating amyloid-induced β-cell death as well as amyloid-induced inhibition of β-cell proliferation. This finding is particularly of importance because growing evidence suggests that amyloid formation may contribute to islet inflammation and elevated islet IL-1β levels in T2D [7, 8, 23–25, 40].

Finally, a recent *in vitro* study suggested that hIAPP may play a dual role in phosphorylation of Erk1/2 and PKB depending on glucose concentration [41]. Visa *et al*. showed that exposure to synthetic hIAPP decreased Erk1/2 and PKB phosphorylation in a transformed β-cell line and wild-type mouse islets cultured with elevated glucose but increased their phosphorylation in normal glucose concentration. However, the latter scenario has low physiological significance because elevated glucose is an important factor that initiates and potentiates hIAPP aggregation *in vivo* and amyloid formation in islets typically occurs in pathologic conditions associated with hyperglycemia. Therefore, in our experimental models, we focused on the effects of hIAPP aggregates on β-cell PKB phosphorylation in elevated glucose to mimic the hyperglycemic condition in diabetic patients.

In this study, we chose quantitative immunohistochemistry approach to assess the β-cell specific changes in IL-1β and phospho-PKB levels because islet lysates contain both amyloid-positive (~10–15%) and amyloid-negative (~85–90%) islets, which makes it difficult to detect β-cell specific protein changes in amyloid-positive islets by Western blot analysis of whole islet lysates. However, it should be noted that quantitative immunohistochemistry has limitations, and unlike Western blot analysis, it is a semi-quantitative rather than a quantitative method. Also, the potential impact of relatively small number of amyloid-positive islets in each condition on data analysis has to be considered in the interpretation of findings.

In summary, our findings suggest that amyloid formation reduces PKB phosphorylation possibly by promoting IL-1β production. Inhibitors of hIAPP aggregation and/or IL-1β signaling may provide a new therapeutic strategy to maintain β-cell mass in T2D and enhance long-term survival of islet grafts in clinical islet transplantation.

## Supporting information

S1 Table Primary and secondary antibodies used for immunolabelling and Western blot. (DOCX)
