# Supplementary material for: Amyloid formation reduces protein kinase B phosphorylation in primary islet β-cells which is improved by blocking IL-1β signaling
Source: PLoS One. 2018 Feb 23;13(2):e0193184. doi: 10.1371/journal.pone.0193184 (PMC5825069; doi:10.1371/journal.pone.0193184)
Supplement: S1 Table — (DOCX) [file pone.0193184.s001.docx]

**S1 Table. Primary and secondary antibodies used for immunolabelling and Western blot.**

**Primary Antibodies**

| **Antibody** | **Anti-insulin** | **Anti-Glucagon** | **Anti-pPKB** | **Anti-tPKB** | **Anti-A11** | **Anti-IL-1β** | **Anti-PCNA** |
| --- | --- | --- | --- | --- | --- | --- | --- |
| **Company** | Dako | Sigma-Aldrich | Cell signaling | Cell signaling | Invitrogen | Santa Cruz | Cell Signaling |
| **Catalogue #** | A0564 | G2654 | 9271 | 4691S | AHB0052 | Sc-7884 | 2586S |
| **Species Ab raised** | Guinea pig | Mouse | Rabbit | Rabbit | Rabbit | Rabbit | Mouse |
| **Antibody type** | Polyclonal | Monoclonal | Polyclonal | Monoclonal | Polyclonal | Polyclonal | Monoclonal |
| **Specificity** | Human, rat, mouse | Human, porcine, dog, rabbit, mouse  rat, guinea pig, cat | Human, rat, mouse, hamster, bovine, dog, pig | Human, rat, mouse, monkey, D. melanogaster | Human, rat, mouse | Human, rat, mouse | Human, rat, mouse, monkey, bovine, pig |
| **Dilution** | 1:750 | 1:1000 | 1:100 (IM)  1:750 (WB) | 1:750 (WB) | 1:400 | 1:100 | 1:250 |

**Secondary Antibodies**

| **Antibody** | **Texas red conjugated anti-guinea pig** | **Alexa 488 conjugated anti-rabbit** | **Alexa 488 conjugated anti-guinea pig** | **Texas red conjugated anti-rabbit** | **AMCA conjugated anti-guinea pig** | **Alexa 594 conjugated anti-mouse** | **Alexa 488 conjugated anti-mouse** |
| --- | --- | --- | --- | --- | --- | --- | --- |
| **Company** | Jackson Laboratories | Molecular Probes | Molecular Probes | Jackson Laboratories | Jackson  Laboratories | Molecular Probes | Molecular Probes |
| **Catalogue #** | 106-076-003 | A-11008 | A-11073 | 711-075-152 | 706-155-148 | A21203 | A-11001 |
| **Animal raised** | Goat | Goat | Goat | Donkey | Donkey | Donkey | Goat |
| **Antibody type** | Polyclonal | Polyclonal | Polyclonal | Polyclonal | Polyclonal | Polyclonal | Polyclonal |
| **Specificity** | Guinea pig | Rabbit | Guinea pig | Rabbit | Guinea pig | Mouse | Mouse |
| **Dilution** | 1:750 | 1:100 | 1:750 | 1:400 | 1:200 | 1:1000 | 1:250 |
